# Targeting Amyloid Pathology in Early Alzheimer’s: The Promise of Donanemab-Azbt

**DOI:** 10.3390/pharmacy13010023

**Published:** 2025-02-08

**Authors:** Nadia Khartabil, Ayda Awaness

**Affiliations:** School of Pharmacy, West Coast University, Anaheim, CA 92801, USA; aawaness@westcoastuniversity.edu

**Keywords:** Kisunla, donanemab-azbt, Alzheimer’s disease, TRAILBLAZER-ALZ 2,3,4,5,6, amyloid, ARIA

## Abstract

Objective: The purpose of this review is to examine the potential role of donanemab-azbt in the treatment and management of early-stage Alzheimer’s disease (AD), with a focus on its efficacy, safety, and clinical relevance based on data from key clinical trials. Data Sources: A comprehensive literature search of PubMed was conducted using relevant keywords such as “donanemab”, “Alzheimer’s disease”, “Kisunla”, “TRAILBLAZER clinical trials”, and “amyloid-related imaging abnormalities (ARIA)”. Additional data were extracted from clinical trial records (clinicaltrials.gov), conference abstracts, and product monographs. Study Selection and Data Extraction: Only English-language studies conducted in human populations were included. Clinical trials and peer-reviewed studies detailing the efficacy, safety, and mechanistic insights of donanemab-azbt were prioritized. Data Synthesis: Key findings from the TRAILBLAZER series of clinical trials highlighted the potential of donanemab-azbt in slowing cognitive and functional decline in early-stage AD: (1) TRAILBLAZER-ALZ (Phase 2): This trial focused on participants with intermediate levels of tau protein. Results demonstrated a statistically significant slowing of cognitive and functional decline. (2) TRAILBLAZER-ALZ 2 (Phase 3): A large-scale, randomized, double-blind, placebo-controlled study confirmed the efficacy of donanemab-azbt in reducing amyloid plaque accumulation and cognitive decline. Key results included a 35% slowing of decline on the Integrated Alzheimer’s Disease Rating Scale (iADRS) and a 36% slowing on the Clinical Dementia Rating-Sum of Boxes (CDR-SB). Additional secondary outcomes showed improvements in activities of daily living and reduced risk of disease progression. (3) TRAILBLAZER-ALZ 3: This ongoing trial is evaluating donanemab’s potential in delaying or preventing Alois Alzheimer in cognitively normal individuals with amyloid plaques, broadening the scope of early intervention strategies. (4) TRAILBLAZER-ALZ 4: A head-to-head comparison with aducanumab revealed superior amyloid plaque clearance with donanemab. (5) TRAILBLAZER-ALZ 5: Currently recruiting, this trial aims to evaluate safety and efficacy across diverse populations with varying tau levels and comorbidities. (6) TRAILBLAZER-ALZ 6 (Phase 3b): This trial investigates modified dosing regimens to reduce ARIA while maintaining efficacy, particularly in populations with genetic risk factors like ApoE ε4 homozygotes. Relevance to Patient Care and Clinical Practice: Donanemab-azbt represents a promising treatment option for patients with early-stage AD. It specifically targets and reduces amyloid beta plaques, a hallmark of the disease, potentially slowing progression and preserving cognitive function. However, its administration requires careful patient selection, including genetic testing for ApoE ε4 status, to mitigate risks of ARIA. Furthermore, the findings emphasize the importance of close monitoring during treatment. Conclusions: Donanemab-azbt offers a new avenue for managing early-stage AD, showing promise in reducing amyloid burden and slowing cognitive decline. While its efficacy and safety have been demonstrated in clinical trials, further research is essential to validate long-term outcomes, assess effectiveness across diverse populations, and refine dosing strategies to minimize side effects. With continued investigation, donanemab-azbt could significantly impact the clinical landscape of AD treatment.

## 1. Introduction

Alzheimer’s disease (AD), the most common cause of dementia, is a progressive neurodegenerative disease characterized by two key pathological features in the central nervous system (CNS): extracellular amyloid plaques derived from amyloid-beta peptides and neurofibrillary tangles formed from aggregated and hyperphosphorylated tau proteins [1]. Tau is a microtubule-associated protein that stabilizes the structural framework of neurons and facilitates intracellular transport. In AD, tau becomes hyperphosphorylated, detaches from microtubules, and aggregates into neurofibrillary tangles, disrupting normal neuronal function. Similarly, amyloid-beta (Aβ) is a peptide formed by the cleavage of the amyloid precursor protein (APP) through beta- and gamma-secretase enzymes. While Aβ is typically regulated and cleared in healthy brains, in AD, its excessive production and impaired clearance lead to the formation of amyloid plaques, a hallmark of the disease. Brain imaging studies in both early and late-onset AD reveal significant disruptions in Aβ homeostasis, which appears to initiate tau pathology. In the early stages, Aβ is believed to facilitate the spread of tau, contributing to cortical neurodegeneration. As the disease progresses, tau neurofibrillary tangles and Aβ plaques accumulate concurrently in the cortical regions of the brain, highlighting a dependency of tau pathology on Aβ levels [2].

Over 55 million people worldwide are living with dementia, with more than 60% residing in low- and middle-income countries. According to the World Health Organization, there are nearly 10 million new cases of dementia every year [2]. In the United States, an estimated 6.9 million Americans aged 65 and older currently have AD [3]. This number is projected to rise to 13.8 million by 2060 unless medical breakthroughs are made to prevent, slow, or cure AD [4]. Clinically, AD is characterized by memory loss, confusion, poor judgment, language disturbance, visual complaints, agitation, withdrawal, and hallucinations. Furthermore, the progress of the disease into the late stage is marked by loss of brain function leading to infection, pneumonia, dehydration, poor nutrition, and possibly death [5]. Research studies have shown that 75–87% of people with AD develop impairments in cough/and swallowing. Dysphagia increases the risk of sarcopenia, lower body mass, and various degrees of malnutrition [6]. This increases the risk of aspiration pneumonia leading to infections and hospitalizations [7].

AD can be broadly categorized into dominantly inherited familial AD (FAD), early-onset AD (EOAD), and late-onset AD (LOAD), each with distinct genetic and clinical characteristics. FAD, a rare form of AD caused by mutations in the amyloid precursor protein (APP), presenilin 1 (PSEN1), or presenilin 2 (PSEN2) genes, accounts for less than 1% of all cases. It typically manifests early, with an average age of onset of around 46.2 years and cases reported as early as the 20s [8].

EOAD is diagnosed in individuals younger than 65 years and, while slightly more common than FAD, comprises fewer than 5% of pathologically confirmed cases. EOAD often presents atypically and follows a more aggressive progression compared to other forms of AD [9]. Additionally, individuals with Down syndrome who have a partial or full trisomy of chromosome 21, which includes the APP gene, almost universally develop AD pathology by age 40. Most exhibit clinical symptoms by age 50, and a majority progress to dementia by age 65 [10,11].

LOAD, the most prevalent form of AD, is generally sporadic but influenced by genetic risk factors. The apolipoprotein E (APOE) gene, particularly the APOE4 allele, plays a significant role in disease risk. A single copy of the APOE4 allele increases the odds of developing AD by approximately threefold, while homozygosity for APOE4 raises the odds twelvefold [11]. The APOE4 allele is also linked to increased susceptibility to vascular dementia, Lewy body dementia, and traumatic brain injury, as well as AD pathology in individuals with Down syndrome [12].

Beyond APOE, additional genetic risk factors for LOAD, including TREM2, ADAM10, and PLD3, have been identified through genome-wide association studies. These genes are implicated in processes such as cholesterol metabolism, immune response, endocytosis, and the regulation of APP and tau proteins, furthering our understanding of AD pathogenesis [13,14]. Ongoing research into these and newly discovered genetic factors continues to offer valuable insights into the mechanisms underlying the development and progression of AD.

Pathologically, the main microscopic feature of extracellular amyloid plaques and intracellular neurofibrillary tangles was first observed over a century ago [15]. Amyloid plaques, initially described by Alois Alzheimer as “miliary foci” are formed by the extracellular accumulation of Aβ40 and Aβ42 peptides resulting from the aberrant processing of amyloid precursor protein by β- and γ-secretases. These processes create an imbalance between production and clearance of amyloid peptides [16]. The resulting 4 kDa peptides fold into beta-pleated sheet structures, which are highly fibrillogenic.

There are numerous types of nonvascular amyloid deposits described, but diffuse plaques and dense core plaques are the most prevalent in AD pathology [17]. Diffuse plaques, which initially form in the neuropil, show weak staining with amyloid-binding dyes such as thioflavin S and Congo red. They often lack argyrophilia on Bodian silver staining and are not associated with significant microglial or astrocytic activation. Dense core plaques, on the other hand, exhibit compact, radiating amyloid deposits that intensely stain with amyloid-specific dyes and are associated with more fibrillogenic forms of Aβ [18].

**Figure 1:**
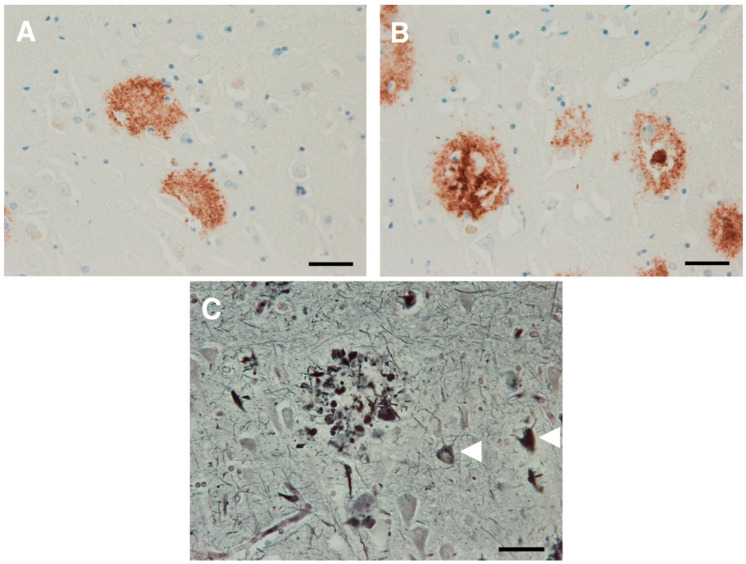
Alzheimer Senile Plaques. Immunohistochemistry of affected Alzheimer’s tissue using antibodies directed against Ab peptides. (**A**): Diffuse plaques (**B**): Dense core (**C**): Neuritic plaques. Neuritic AD plaques are readily observed using Bielchowsky silver staining as shown above pointed by the white triangles. Image courtesy to *Molecular Neurodegeneration* volume 14, Article number: 32 (2019) [15].

A subset of dense core plaques, referred to as neuritic plaques (NPs), contains tau-positive dystrophic neurites and is linked with synaptic loss, activated microglia, and reactive astrocytes [19]. While diffuse plaques generally lack neuritic components, diffuse neuritic plaques may appear in advanced stages of AD. There is ongoing debate as to whether diffuse plaques represent early stages in the development of neuritic plaques or are part of pathological aging. Additionally, plaques exclusively composed of dense cores without neuritic elements, termed “burnt-out plaques”, are often observed in later stages. Neuritic plaques with dense amyloid and tau-positive neurites are believed to have the strongest association with neuronal loss and cognitive decline in AD [20]. Figure 1A–C below represent diffuse plaques, dense core plaques, and cored neuritic plaques described above.

We shed light on the different types of plaques due to the fact that there are new studies that have identified different roles of these plaques in AD. According to a study using animal models, it was noted that fewer dense core plaques seem to be more detrimental to the disease progression [21].

### 1.1. AD Treatment Landscape

The treatment landscape for AD has evolved significantly over the past few decades. Initially, therapeutic options were limited to cholinesterase inhibitors (e.g., donepezil, rivastigmine) and the NMDA receptor antagonist memantine, which provided modest symptomatic relief without altering disease progression [22]. Recent advancements have introduced anti-amyloid monoclonal antibodies designed to target amyloid-beta plaques, a hallmark of AD pathology. Aducanumab was the first of these agents to receive FDA approval, aiming to reduce amyloid-beta accumulation in the brain. Subsequent developments include lecanemab and donanemab, both of which have demonstrated efficacy in slowing cognitive decline in early AD by targeting amyloid-beta aggregates [23].

Lecanemab was developed by Eisai and Biogen Inc. Tokoyo, Japan. and was approved by the Food and Drug Administration (FDA) in July 2023 and donanemab-azbt which was developed by Eli Lilly and Company, Indianapolis, IN was FDA approved in July 2024 [24,25].

### 1.2. Mechanism of Action

Donanemab and lecanemab are both humanized monoclonal antibodies targeting Aβ proteins. Their mechanism of action differs in terms of the specific forms of Aβ they target and their subsequent effects.

Donanemab-azbt is a humanized immunoglobulin-1 (IgG1) monoclonal antibody that binds specifically to an N-terminal pyroglutamate-modified form of amyloid-beta at position 3 (pGlu3-Aβ). Upon binding to the modified Aβ plaques, donanemab facilitiates their removal through microglial-mediated phagocytosis, a process where immune cells in the brain engulf and degrade the plaques [26].

Lecanemab targets soluble amyloid beta protofibrils, which are the precursors to the insoluble amyloid plaques. By binding to these protofibrils, lecanemab promotes their clearance, thus reducing the formation of amyloid plaques in the brain [27].

Compared to lecanemab and donanemab, aducanumab involves selectively binding to both soluble and insoluble forms of Aβ aggregates, including oligomers and fibrils, facilitating their removal and potentially mitigating neurodegeneration [28].

In summary while both donanemab and lecanemab aim to clear the Aβ plaques present in AD, they do so by targeting different forms of Aβ: donanemab focuses on established plaques, whereas lecanemab targets soluble protofibrils to prevent plaque formation [29].

**Figure 2:**
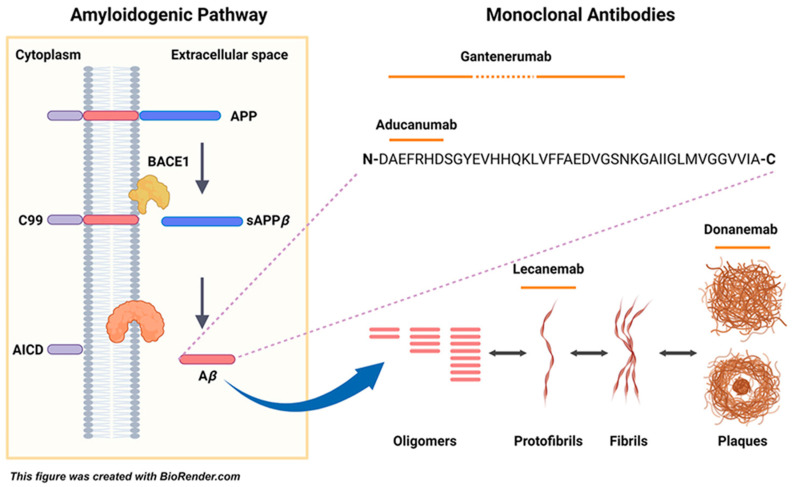
Molecular Targets of Anti-Amyloid Monoclonal Antibodies. Processing of APP and production of Aβ. APP is initially cleaved by α-secretase in the Non-amyloidogenic pathway, yielding two fragments: sAPPα and C83. The late is cleaved by the γ-secretase complex, creating the p3 and AICD peptides. In the Amyloidogenic pathway, β-secretase (BACE1) cleaves APP to produce the sAPPβ and C99 fragments. A linear epitope formed by the amino acids 2–7 of Aβ increases aducanumab’s affinity towards aggregates of fibrils Lecanemab recognizes Aβ protofibrils with much higher affinity than monomers. Credit to: Acta Pharmaceutica Sinica B Volume 14, Issue 7, July 2024, Pages 2795–2814 [30].

Please refer to Figure 2 for an illustration of the mode of action.

## 2. Pharmacokinetics and Pharmacodynamics

The pharmacokinetic (PK) profile of donanemab was characterized through a population PK analysis involving participants with AD. This analysis incorporated data from individuals with mild cognitive impairment or mild to moderate dementia due to AD from a phase Ib study, as well as participants with early symptomatic AD from the TRAILBLAZER-ALZ study [31].

The analysis revealed that donanemab has a terminal elimination half-life of approximately 11.8 days. Body weight and antidrug antibody titer were found to impact donanemab exposure; however, these factors did not significantly affect the pharmacodynamic response. Maintaining a donanemab serum concentration above 4.43 μg/mL was associated with amyloid plaque reduction. The time to achieve amyloid plaque clearance varied depending on baseline amyloid levels, with higher baseline levels associated with fewer participants achieving amyloid clearance. The majority of participants achieved amyloid clearance by 52 weeks of treatment [31].

No clinical studies have been conducted to evaluate the pharmacokinetics of donanemab in patients with renal or hepatic impairment. However, due to its degradation pathway, dose adjustments are not necessary for patients with renal or hepatic impairment [32].

Additionally, the analysis indicated that APOE ε4 carriers were four times more likely than noncarriers to experience amyloid-related imaging abnormalities with edema or effusions (ARIA-E) by 24 weeks, irrespective of donanemab serum exposure.

Dosing and Administration: The treatment regimen includes an initial dose of 700 mg administered via intravenous infusion every 4 weeks for 3 doses, followed by 1400 mg IV every 4 weeks until amyloid plaques are significantly reduced on amyloid PET imaging. Infusions are delivered over 30 min, and dilution is performed with normal saline (NS) to a final concentration of 4 to 10 mg/mL. Based on the package insert, once the medication is diluted, the solution should be used immediately, or it may be stored in the refrigerator at 2 °C to 8 °C (36 °F to 46 °F) for up to 72 h, or at room temperature (20 °C to 25 °C [68 °F to 77 °F]) for up to 12 h including the duration of the infusion [33].

## 3. Efficacy and Clinical Trials

The efficacy and safety of donanemab-azbt was assessed in a series of clinical trials. Below is an expanded discussion of these trials.

TRAILBLAZER-ALZ: The TRAILBLAZER-ALZ phase 2 study targeted individuals with early symptomatic AD, particularly those with intermediate levels of tau protein accumulation in the brain. The study demonstrated that donanemab treatment led to a reduction in the rate of cognitive and functional decline [34].

TRAILBLAZER-ALZ 2: was a large-scale, randomized, double-blind, placebo-controlled trial that assessed the safety and efficacy of donanemab. Participants were stratified based on their tau protein levels. The study’s primary analysis population (*n* = 1182), composed of individuals with intermediate tau levels and clinical symptoms of AD, showed significant findings. In this group, the primary endpoint, the Integrated Alzheimer’s Disease Rating Scale (iADRS), demonstrated a 35% reduction in the rate of decline (*p* < 0.0001). Additionally, a key secondary endpoint, the Clinical Dementia Rating-Sum of Boxes (CDR-SB), indicated a 36% reduction in decline (*p* < 0.0001) over an 18-month period. Further prespecified secondary analyses revealed that 47% of participants receiving donanemab experienced no decline on the CDR-SB at one year, compared to 29% in the placebo group (*p* < 0.001). Moreover, 52% of participants in the donanemab group completed the treatment course by one year, with 72% completing it by 18 months, primarily due to achieving amyloid plaque clearance. At 18 months, individuals on donanemab showed 40% less decline in performing activities of daily living (as assessed by the Alzheimer’s Disease Cooperative Study–Instrumental Activities of Daily Living Inventory or ADCS-iADL, *p* < 0.0001). Finally, donanemab-treated participants had a 39% lower risk of progressing to the next stage of the disease compared to those on placebo (measured by the CDR-Global Score, Hazard Ratio = 0.61, *p* < 0.001) [35].

This study was followed by the ongoing TRAILBLAZER-ALZ 3.

TRAILBLAZER-ALZ 3: aims to evaluate the potential to delay or prevent AD in cognitively normal individuals with amyloid plaques but no symptoms. It is part of a broader effort to investigate early intervention strategies. The trial will provide crucial data on the efficacy of amyloid-targeting treatments in the preclinical phase of AD. TRAILBLAZER-ALZ 3 highlights the growing emphasis on early detection and intervention in AD management. If successful, it would support the use of donanemab not only as a treatment for early symptomatic AD but also as a preventive therapy in high-risk individuals. This could shift clinical practice toward more proactive management strategies, emphasizing biomarker screening and early therapeutic intervention to delay or prevent the onset of symptoms, potentially reducing the overall burden of AD [36].

### 3.1. TRAILBLAZER-ALZ 4

Donanemab was studied in a head-to-head trial with aducanumab in TRAILBLAZER-ALZ 4 where 37.9% donanemab-treated vs. 1.6% aducanumab-treated participants achieved amyloid clearance (*p* < 0.001). In the intermediate tau subpopulation, 38.5% donanemab-treated vs. 3.8% aducanumab-treated participants achieved amyloid clearance (*p* = 0.008). These results suggest that donanemab may be more effective at clearing amyloid plaques in AD patients, potentially leading to better clinical outcomes [37].

### 3.2. TRAILBLAZER-ALZ 5

TRAILBLAZER-ALZ 5 focuses on evaluating safety and efficacy in broader populations, including those with varying tau levels and comorbidities. It is an ongoing Phase 3, double-blind, placebo-controlled clinical trial designed to assess the safety and efficacy of donanemab in individuals with early symptomatic AD, specifically those in the prodromal stage or with mild dementia due to AD, who exhibit brain tau pathology. The trial aims to enroll participants aged 60 to 85 years who have demonstrated gradual and progressive changes in memory function over at least six months, possess a Mini-Mental State Examination (MMSE) score between 20 and 28, and have confirmed tau pathology via brain imaging [38].

Participants are randomly assigned to receive either intravenous infusions of donanemab or a placebo. The study’s primary objective is to evaluate the efficacy of donanemab in slowing cognitive and functional decline, as measured by the Integrated Alzheimer’s Disease Rating Scale (iADRS). Secondary objectives include assessments of safety, changes in amyloid and tau levels, and other cognitive and functional measures.

TRAILBLAZER-ALZ 6 (Phase 3b) results were presented in 2024. It highlighted a modified titration regimen that reduced amyloid-related imaging abnormalities with edema/effusion (ARIA-E) while maintaining plaque removal efficacy. This was particularly effective for individuals with genetic risk factors (APOE4 homozygotes). Interim results from TRAILBLAZER-ALZ 6 indicated that the modified titration dosing regimen led to a significant reduction in the occurrence of ARIA-E compared to the standard dosing regimen. Specifically, there was a 41% reduction in the relative risk of ARIA-E in the modified dosing group. Safety profiles were comparable across both groups, with no new safety signals identified. The frequency of infusion-related reactions in the modified titration arm was similar to that of the standard dosing arm. The findings from TRAILBLAZER-ALZ 6 suggest that implementing a modified titration dosing regimen of donanemab may enhance the safety profile of the treatment by reducing the risk of ARIA-E, without compromising its efficacy [39].

A summary of the clinical trials is listed in Table 1.

## 4. Safety and Tolerability

Donanemab-azbt common adverse reactions included ARIA-E or Amyloid Related Imaging Abnormalities with Edema, ARIA-H (Amyloid Related Imaging Abnormalities with Hemosiderin Deposition) microhemorrhage, ARIA-H with Superficial Siderosis, allergic and infusion related reactions, and headache. The treatment group experienced a 10% or higher incidence than the placebo group. This drug is contraindicated in patients with a known severe hypersensitivity to donanemab-azbt or any of its excipients. The drug carries a black box warning regarding ARIA (Amyloid-Related Imaging Abnormalities), which can manifest as either edema (ARIA-E) or hemosiderin deposition (ARIA-H). This typically occurs early in treatment and is often asymptomatic, but it can be serious and life-threatening. Significant intracerebral hemorrhages greater than 1 cm have been observed in patients using this medication. To prevent this, providers should perform APOE ε4 testing on patients before starting treatment with donanemab, as patients who are APOE ε4 homozygotes—about 15% of AD patients—are at a higher risk of developing ARIA, including symptomatic, serious, and severe radiographic ARIA, compared to heterozygotes and non-carriers [44].

Due to the risk of developing ARIA, it is recommended to obtain a baseline brain magnetic resonance imaging (MRI) before initiating treatment, and before the 2nd, 3rd, 4th and 7th infusion. Making recommendations for patients who develop ARIA depends on clinical symptoms and radiographic severity [45].

Other than ARIA, studies reported infusion-related reactions in 9% of patients treated with donanemab compared to 0.5% of patients on placebo. These reactions occurred mainly within the first 4 infusions and the signs included: chills, erythema, headaches, nausea/vomiting, difficulty breathing, sweating, and low blood pressure. The manufacturer recommends reducing or discontinuing the infusion if clinically indicated. Pre-treatment with acetaminophen, antihistamines, or corticosteroids may be considered [46].

## 5. Comparison with Existing FDA-Approved Medications

Cholinesterase inhibitors include donepezil approved for all stages of AD, galantamine, and rivastigmine for mild to moderate AD. These drugs work by increasing the amount of acetylcholine to improve the cognitive symptoms. NMDA receptor antagonist such as memantine is approved for moderate to severe AD. This drug helps to reduce the excitotoxicity related to an increased activation of glutamate receptors. The limitations of these two classes of drugs are that they only help to manage the symptoms of the disease, without tackling the underlying causes of AD. In addition, cholinesterase inhibitors’ side effects include sleep disturbances, nausea, vomiting, diarrhea, muscle weakness, headache, and dizziness [47]. NMDA receptor antagonist side effects include confusion, headache, dizziness, and constipation. The above small molecule drugs target either acetylcholine levels or excitotoxicity, while the new recently developed amyloid-targeting therapies address amyloid plaques for potentially disease modifying therapies.

Amyloid-targeted therapies include lecanemab, aducanumab, and the newly approved donanemab. These drugs aim to inhibit the aggregation of amyloid-beta plaques in the brain. Among these, aducanumab was removed from the market in November 2024 based on a business decision by Biogen [48].

Currently, no direct head-to-head clinical trials are comparing small-molecule AD drugs like memantine and donepezil with monoclonal antibodies such as donanemab or lecanemab. However, indirect comparisons through meta-analyses and systematic reviews provide some insights into their relative efficacy and safety profiles.

Clinical studies showed that donanemab and lecanemab were significantly superior to placebo in efficacy in Clinical Dementia Rating sum of boxes (CDR-SB) [49]. A systematic review conducted by Terao, et al. stated the incidence of ARIA-E to be 36% and 35% in the two included trials of aducanumab, 12.6% and 9.9% in the two included trials of lecanemab, 26.7% and 24% in the two included trials of donanemab. ARIA-H: 19% and 20% in the two included trials of aducanumab, 17.3% and 6.8% in the two included trials of lecanemab, 22.1% and 19.7% in the two included trials of donanemab. Please refer to Table 2 for a comparison of donanemab and lecanemab [49].

## 6. Place in Therapy and Clinical Recommendations

Donanemab-azbt is a promising treatment recommended for adults’ patients with early symptoms of AD, including mild cognitive impairment (MCI) or mild dementia (mini-mental state examination (MMSE) of 21–26 with confirmed abnormal amyloid. This drug works by removing the amyloid beta plaques (a key feature in AD) that formed in the brain of AD patients; therefore, adequately slowing functional and cognitive declines. In therapy, donanemab-azbt can be considered as first-line for this particular category of patients. It is recommended that candidates for this drug have no underlying bleeding condition, no transient ischemic attack (TIA), stroke, seizure in the past 12 months, no malignancies within 3 years of screening, and no underlying conditions that may cause cognitive impairment. Other underlying conditions that may cause cognitive impairment include vitamin B12 deficiency, depression (except those with Geriatric Depression Scale or GDS > 7), and substance abuse within 2 years. Since there is a need for brain MRI, patients eligible for this medication should have no brain magnetic resonance imaging (MRI) contraindication [51].

### Economic and Quality-of-Life Considerations

Table 3 below from Eli Lilly and Company represents the total cost of treatment with donanemab-azbt after 6, 12, and 18 months. Out-of-pocket expenses like the duration of treatment, monthly clinical visits for IV infusion, imaging (PET scan, MRI), and any additional clinic fees will vary based on each patient’s insurance plan. The cost of a single vial is $695.65. The first 3 doses consist of 2 vials (700 mg) followed by 4 vials (1400 mg) doses after.

## 7. Conclusions

Donanemab-azbt is a monoclonal antibody used for early stages of AD. Clinical studies have shown that donanemab is effective in slowing cognitive and functional decline by reducing and targeting amyloid-beta plaques, which are essential to the pathophysiology of AD [53]. According to the findings of the TRAILBLAZER-ALZ 2 trial, donanemab-azbt can dramatically lower the buildup of amyloid plaque, which may change how the disease progresses for patients who are still in early stages. Donanemab-azbt carries a black box warning for ARIA therefore, genotype testing for APOE ε4 along with monitoring are recommended. Long-term research on the efficacy and safety of donanemab-azbt is ongoing and is essential to evaluate future directions in treatment recommendations.

## Figures and Tables

**Table 1:** Summary of Donanemab Clinical Trials.

Key Clinical Trials Name [34,35,40,41,42,43] Drug Studied	Number of Participants	Age Range (yr)	Phase	Location	Identifier
TRAILBLAZER-EXT Donanemab	94	60–90	II	Canada USA	NCT04640077
TRAILBLAZER-ALZ Donanemab	272	60–85	II	Canada USA	NCT03367403
TRAILBLAZER-ALZ 2 Donanemab/Placebo	1736	60–85	III	Global	NCT04437511
TRAILBLAZER-ALZ 3 Donanemab/Placebo	2196	65–80	III	Japan, Puerto Rico, USA	NCT05026866
TRAILBLAZER-ALZ 4 Donanemab/Aducanumab	148	50–85	III	USA	NCT05108922
TRAILBLAZER-ALZ 5 Donanemab/Placebo	Actively recruiting	60–85	III	Global	NCT05508789
TRAILBLAZER-ALZ 6 Donanemab/Placebo	800	60–85	III	Global	NCT05738486

**Table 2:** Comparison of the Amyloid Target Therapy.

Drug Name [25,50]	Dosage	Administration	Frequency	Cost	Mechanism of Action
Donanemab-azbt (Kisunla)	Initial: 700 mg every 4 weeks for 3 doses; Maintenance: 1400 mg every 4 weeks	IV infusion over 30 min	Once every 4 weeks	$32,000 per year	Humanized monoclonal antibody targeting insoluble N-truncated pyroglutamate amyloid beta
Lecanemab (Leqembi)	10 mg/kg body weight	IV infusion over 1 h	Once every 2 weeks	$26,500 per year	Humanized monoclonal antibody binding to soluble and insoluble toxic amyloid-beta protofibrils

**Table 3:** Cost of treatment with Donanemab-azbt in the United States [52].

Length of Treatment	6 Months	12 Months	18 Months
30 min infusion	6	13	19
Course of therapy cost	$12,522	$32,000	$48,696
